# Machine learning algorithms assist early evaluation of enteral nutrition in ICU patients

**DOI:** 10.3389/fnut.2023.1060398

**Published:** 2023-04-14

**Authors:** Ya-Xi Wang, Xun-Liang Li, Ling-Hui Zhang, Hai-Na Li, Xiao-Min Liu, Wen Song, Xu-Feng Pang

**Affiliations:** ^1^Department of Hospital-acquired Infection Control, The Affiliated Hospital of Qingdao University, Qingdao, Shandong, China; ^2^Department of Nephrology, The Second Hospital of Anhui Medical University, Hefei, Anhui, China; ^3^School of Nursing, Qingdao University, Qingdao, Shandong, China; ^4^Department of Nephrology, The Affiliated Hospital of Qingdao University, Qingdao, Shandong, China; ^5^Department of Critical Care Medicine, The Affiliated Hospital of Qingdao University, Qingdao, Shandong, China; ^6^Department of Endoscopy, The Affiliated Hospital of Qingdao University, Qingdao, Shandong, China

**Keywords:** enteral nutrition, intensive care unit, machine learning, initiation, prediction

## Abstract

**Background:**

This study applied machine learning (ML) algorithms to construct a model for predicting EN initiation for patients in the intensive care unit (ICU) and identifying populations in need of EN at an early stage.

**Methods:**

This study collected patient information from the Medical Information Mart for Intensive Care IV database. All patients enrolled were split randomly into a training set and a validation set. Six ML models were established to evaluate the initiation of EN, and the best model was determined according to the area under curve (AUC) and accuracy. The best model was interpreted using the Local Interpretable Model-Agnostic Explanations (LIME) algorithm and SHapley Additive exPlanation (SHAP) values.

**Results:**

A total of 53,150 patients participated in the study. They were divided into a training set (42,520, 80%) and a validation set (10,630, 20%). In the validation set, XGBoost had the optimal prediction performance with an AUC of 0.895. The SHAP values revealed that sepsis, sequential organ failure assessment score, and acute kidney injury were the three most important factors affecting EN initiation. The individualized forecasts were displayed using the LIME algorithm.

**Conclusion:**

The XGBoost model was established and validated for early prediction of EN initiation in ICU patients.

## 1. Introduction

The nutritional status has a strong association with clinical outcomes in critically ill patients. Malnutrition can lead to more complications, difficulty in ventilator discontinuation, prolonged length of stay in intensive care unit (ICU), and increased readmission rate and mortality (1). Enteral nutrition (EN) refers to a nutrition therapy that foods for special medical purposes are administered *via* the gastrointestinal tract. EN should be preferred when gastrointestinal function allows (2, 3).

EN is an important treatment method for patients in the ICU. EN promotes the recovery of intestinal function and reduces the consumption of skeletal muscle. EN can also decrease the incidence of infection and mortality and shorten the time of hospitalization and medical expenses (4, 5). The American Society for Parenteral and Enteral Nutrition recommends early use of EN in appropriate critically ill patients (6). However, the proportion of EN feeding is suboptimal (7). Therefore, there is a need to identify high-risk patients who will benefit from early EN, which may aid clinicians in decision-making and improve the outcome of critically ill patients.

At present, EN initiation largely depends on physicians’ awareness. Machine learning (ML) is a subset of artificial intelligence (AI) with the ability to process complex data and quantify the risk of event occurrence (8). It includes logistic regression (LR), support vector machine (SVM), decision tree (DT), κ-nearest neighbor (KNN), random forest (RF), and extreme gradient boost (XGBoost), etc. (8). ML algorithms have been widely used in clinical nutrition and critical care (9–11), including diet pattern analysis, disease diagnosis, and prognosis prediction. However, it has not been applied to initiate EN in ICU patients. Accordingly, we aimed to develop a model to predict EN initiation for patients in an ICU setting. This model can assist early identification of populations in need of EN, and provide a basis for physicians’ decision-making to initiate EN. Furthermore, we wished this model can guide the development of standard nutrition protocol and the improvement the nutrition therapy.

## 2. Methods

### 2.1. Data source

The information was collected from the database of Medical Information Mart for Intensive Care IV (MIMIC-IV). About 76,000 ICU admissions were included in MIMIC-IV, authorized by the Institutional Review Boards at Beth Israel Deaconess Medical Center (2001-P-001699/14) and the Massachusetts Institute of Technology (No. 0403000206). This is a freely available, retrospective, singer-center database where patients offered consent for their data to be accessed (12). The ethical approval statement was waived because patients were not directly involved in this study. One of our members was responsible for data extraction from this database (certification number 35970146). This study adhered to the tenets of the Declaration of Helsinki in 2013 and was conducted per the Transparent Reporting of a multivariable prediction model for Individual Prognosis or Diagnosis (TRIPOD) Statement (13).

### 2.2. Study population

We enrolled all 18-year-old and older adults from the MIMIC IV database. Patients who had multiple ICU admissions were excluded.

### 2.3. Data extraction and processing

Clinical information was gathered from the MIMIC-IV database, including patient demographics, comorbidities, vital signs, laboratory results, treatments, and illness severity scores. Age, sex, weight, and ethnicity were collected as demographic characteristics. Comorbidities in our study included diabetes, congestive heart failure, myocardial infarction, cerebrovascular disease, peripheral vascular disease, chronic lung disease, liver disease, renal disease, tumor, dementia, rheumatic disease, intestinal fistula, short bowel syndrome, pancreatitis, abdominal hypertension, peptic ulcer disease, paraplegia, acute kidney injury (AKI), and acquired immune deficiency syndrome. The Implementation of the International Statistical Classification of Disease and Related Health Problems, 10th Revision coding systems were used to define these conditions (14). Within the first 24 h of their ICU stay, we collected averages of vital signs such as mean arterial pressure (MAP), heart rate, body temperature, respiratory rate, and oxygen saturation (SpO_2_). We extracted the maximum values in the initial 24 h admitted to ICU for laboratory findings (Supplementary Table S1). For treatment, EN route (percutaneous endoscopic gastrostomy (PEG), percutaneous endoscopic jejunostomy (PEJ), Nasogastric tube, and Nasointestinal tube), dialysis, vasopressors, and mechanical ventilation were chosen within 24 h of ICU admission. Simplified Acute Physiology Score II for severity scores of illness and Sequential Organ Failure Assessment (SOFA) were gathered within the initial 24 h after ICU admission. The missing data of all valuables in this study were below 20% and dealt with by the multiple imputation (MI) method (Supplementary Table S2). The main process of MI begins by creating multiple data sets, which are then analyzed separately to obtain a set of parameter estimate values. Finally, all estimates is combined and evaluated to get the plausible estimates for the missing data (15). In this study, we perform MI using the MICE package of R software, whose default interpolation method is multiple imputation by chained equations. EN is defined as nutritional support *via* a nasoduodenal or nasogastric tube during the ICU (16). For each patient, the protein intake was 1.2–2.0 g/kg/day and lower energy intake should be preferred in the early stage (6). The optimal nutrition formula of patients was confirmed based on nutritional status and primary diseases.

### 2.4. Statistical analysis

The continuous variables of normal distribution were expressed by means ± standard deviations, and Student’s t-test was utilized to assess differences between groups. The continuous data of skew distribution were described by median and interquartile range (IQR), and the Mann–Whitney U test was employed to compare the two groups. Categorical data were described by frequency and percentage, and the comparison between two groups was performed by Chi-square test or Fisher’s exact test. All data were statistically analyzed using R software (Version 4.2.3) and Python (Version 3.9.12). A two-sided *p* value below 0.05 was perceived as statistically significant.

### 2.5. Machine learning models

We randomized all the patients into two parts, a training set (80%) and a validation set (20%). The comparisons of parameters between the two sets were presented in Supplementary Table S3. We tried to construct and validate the models using LR, SVM, DT, KNN, RF, and XGBoost. We initially used default hyper-values to generate an initial model, and then used grid search and ten-fold cross-validation to find the ideal parameters for each model. We computed accuracy, sensitivity, specificity, and the area under curve (AUC) and plotted the calibration curve and decision curve analysis (DCA) for the validation cohort to assess the predictive performance of models. We confirmed the final model with the optimized performance according to the AUC and accuracy. The difference of AUCs was compared with DeLong test. Furthermore, SHapley Additive exPlanation (SHAP) values were intended to enhance the clarity and interpretability of the best model (17). We used the SHAP summary plot to depict the rank of the predictors attributed to the model. The SHAP dependency graph was used to analyze the importance of a single feature affecting the model output. Finally, the Local Interpretable Model-Agnostic Explanations (LIME) algorithm was implemented to illustrate the impact of these attributes on the best model for each patient (18).

## 3. Results

### 3.1. Baseline characteristics

**Figure 1:**
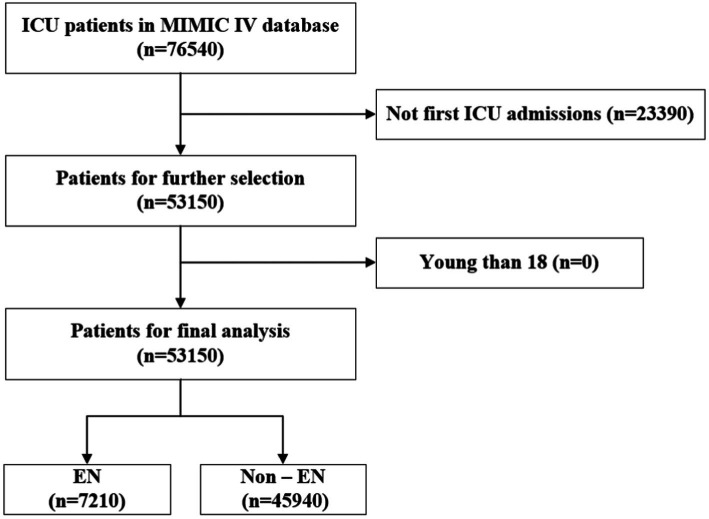
Patient selection from the MIMIC IV database.

A total of 76,540 participants were screened for eligibility. Twenty-three thousand three hundred and ninety patients were eliminated due to numerous ICU admissions (including only the first admission for analysis), and 53,150 patients were recruited (Figure 1). The utilization rate of EN was 13.57% (7,210/53150). The median age of these patients was 66.76 (IQR, 54.49–78.24) years, and 43.9% (23,353/22360) were female. Diabetes (14,613/53150, 27.50%), congestive heart failure (12,622/53150, 23.70%), and chronic lung disease (12,398/53150, 23.30%) were the top three comorbidities. Baseline characteristics comparisons between groups are summarized in Table 1.

**Table 1:** Baseline characteristics between EN group and non-EN group.

Variables	Total (*N* = 53,150)	Non – EN (*n* = 45,940)	EN (*n* = 7,210)	*p-* value
Age (years)	66.76 (54.49–78.24)	66.81 (54.50–78.39)	66.56 (54.48–77.57)	0.027
Sex, male, n (%)	29,797 (56.1)	25,709 (56.0)	4,088 (56.7)	0.246
Weight (kg)	78.45 (65.90–93.10)	78.40 (66.00–93.00)	78.90 (65.50–95.30)	0.016
Ethnicity, n (%)				<0.001
White	35,668 (67.1)	31,318 (68.2)	4,350 (60.3)	
Black	4,874 (9.2)	4,238 (9.2)	636 (8.8)	
Other	12,608 (23.7)	10,384 (22.6)	2,224 (30.8)	
Myocardial infarct, n (%)	8,531 (16.1)	7,468 (16.3)	1,063 (14.7)	0.001
Congestive heart failure, n (%)	12,622 (23.7)	10,717 (23.3)	1905 (26.4)	<0.001
Peripheral vascular disease, n (%)	5,820 (11.0)	5,022 (10.9)	798 (11.1)	0.746
Cerebrovascular disease, n (%)	8,539 (16.1)	6,596 (14.4)	1943 (26.9)	<0.001
Dementia, n (%)	1930 (3.6)	1,612 (3.5)	318 (4.4)	<0.001
Chronic pulmonary disease, n (%)	12,398 (23.3)	10,515 (22.9)	1883 (26.1)	<0.001
Rheumatic disease, n (%)	1717 (3.2)	1,506 (3.3)	211 (2.9)	0.125
Liver disease, n (%)	5,766 (10.8)	4,546 (9.9)	1,220 (16.9)	<0.001
Peptic ulcer disease, n (%)	1,457 (2.7)	1,243 (2.7)	214 (3.0)	0.219
Intestinal fistula, n (%)	170 (0.3)	126 (0.3)	44 (0.6)	<0.001
Short bowel syndrome, n(%)	40 (0.1)	30 (0.1)	10 (0.1)	0.06
Acute pancreatitis, n (%)	907 (1.7)	627 (1.4)	280 (3.9)	<0.001
Abdominal Hypertension, n (%)	1,144 (2.2)	553 (1.2)	591 (8.2)	<0.001
Diabetes, n (%)	14,613 (27.5)	12,582 (27.4)	2031 (28.2)	0.172
Paraplegia, n (%)	2,748 (5.2)	1799 (3.9)	949 (13.2)	<0.001
Renal disease, n (%)	9,386 (17.7)	8,050 (17.5)	1,336 (18.5)	0.039
Tumor, n (%)	7,723 (14.5)	6,678 (14.5)	1,045 (14.5)	0.938
Aids, n (%)	284 (0.5)	237 (0.5)	47 (0.7)	0.166
AKI, n(%)	29,551 (55.6)	23,280 (50.7)	6,271 (87.0)	<0.001
Sepsis, n (%)	23,901 (45.0)	17,830 (38.8)	6,071 (84.2)	<0.001
Heart rate (beats/min)	82.83 (73.25–94.16)	82.40 (73.00–93.42)	86.42 (75.39–98.88)	<0.001
MAP (mmHg)	77.52 (71.27–85.61)	77.54 (71.28–85.65)	77.41 (71.21–85.37)	0.345
Respiratory rate (beats/min)	18.38 (16.39–20.96)	18.24 (16.31–20.72)	19.50 (17.08–22.47)	<0.001
Body temperature (°C)	36.81 (36.59–37.07)	36.79 (36.58–37.03)	36.99 (36.67–37.39)	<0.001
SpO2 (%)	97.07 (95.69–98.36)	97.00 (95.65–98.25)	97.60 (96.00–98.95)	<0.001
Hematocrit (%)	35.20 (31.00–39.60)	35.20 (31.00–39.50)	35.50 (30.90–40.20)	0.001
Hemoglobin (g/dL)	11.70 (10.20–13.20)	11.60 (10.20–13.20)	11.70 (10.10–13.30)	0.572
Platelets (K/uL)	210.00 (158.00–275.00)	210.00 (158.00–274.00)	215.00 (156.00–282.00)	0.042
WBC (K/μL)	12.30 (8.80–16.70)	12.00 (8.70–16.40)	13.90 (10.10–19.00)	<0.001
Bicarbonate (mmol/L)	24.00 (22.00–27.00)	24.00 (22.00–27.00)	24.00 (21.00–27.00)	<0.001
Anion gap (mEq/L)	15.00 (13.00–18.00)	15.00 (13.00–18.00)	16.00 (14.00–19.00)	<0.001
BUN (mg/dL)	19.00 (14.00–30.00)	19.00 (13.00–29.00)	23.00 (15.00–37.00)	<0.001
Serum calcium (mg/dL)	8.60 (8.10–9.00)	8.60 (8.10–9.00)	8.50 (8.10–9.00)	0.003
Serum sodium (mEq/L)	140.00 (137.00–142.00)	140.00 (137.00–142.00)	141.00 (138.00–144.00)	<0.001
Serum chloride (mEq/l)	106.00 (102.00–109.00)	106.00 (102.00–109.00)	106.00 (103.00–110.00)	<0.001
Serum potassium (mEq/L)	4.40 (4.00–4.80)	4.40 (4.00–4.80)	4.40 (4.00–5.00)	<0.001
Creatinine (mg/dL)	1.00 (0.80–1.40)	1.00 (0.80–1.40)	1.10 (0.80–1.80)	<0.001
Glucose (mg/dL)	137.00 (113.00–178.00)	135.00 (112.00–173.00)	156.00 (126.00–205.00)	<0.001
INR	1.30 (1.10–1.50)	1.30 (1.10–1.50)	1.30 (1.10–1.60)	<0.001
PT (s)	14.00 (12.40–16.60)	14.00 (12.40–16.40)	14.30 (12.60–17.90)	<0.001
PTT (s)	31.30 (27.50–40.00)	31.20 (27.50–39.40)	32.30 (27.70–44.30)	<0.001
eGFR(ml/min/1.73 m^2^)	1.01 (0.81–1.06)	1.01 (0.81–1.06)	1.01 (0.81–1.06)	0.025
Dialysis, n (%)	1,668 (3.1)	1,228 (2.7)	440 (6.1)	<0.001
Vasopressors use, n (%)	2004 (3.8)	1,324 (2.9)	680 (9.4)	<0.001
Parental Nutrition, n(%)	885 (1.7)	470 (1.0)	415 (5.8)	<0.001
Mechanical ventilation, n (%)	38,366 (72.2)	31,872 (69.4)	6,494 (90.1)	<0.001
PEG, n (%)	934 (1.8)	174 (0.4)	760 (10.5)	<0.001
PEJ, n (%)	66 (0.1)	23 (0.1)	43 (0.6)	<0.001
Nasogastric tube, n (%)	164 (0.3)	113 (0.2)	51 (0.7)	<0.001
Nasointestinal tube, n (%)	47 (0.1)	21 (0.0)	26 (0.4)	<0.001
SOFA score	4.00 (2.00–6.00)	3.00 (2.00–6.00)	7.00 (4.00–11.00)	<0.001
SAPS II score	33.00 (25.00–42.00)	32.00 (24.00–40.00)	40.00 (31.00–51.00)	<0.001

Numbers are presented as median (interquartile range) or numbers (percentages). EN, enteral nutrition; Aids, acquired immune deficiency syndrome; AKI, acute kidney injury; MAP, mean arterial pressure; SpO2, oxygen saturation; WBC, white blood cell; BUN, blood urea nitrogen; INR, international normalized ratio; PT, prothrombin time; PTT, partial thromboplastin time; eGFR, estimated glomerular filtration rate; PEG, percutaneous endoscopic gastrostomy; PEJ, Percutaneous Endoscopic Jejunostomy; SAPS II, Simplified Acute Physiology Score II; SOFA, sequential organ failure assessment.

### 3.2. Model development and validation

**Figure 2:**
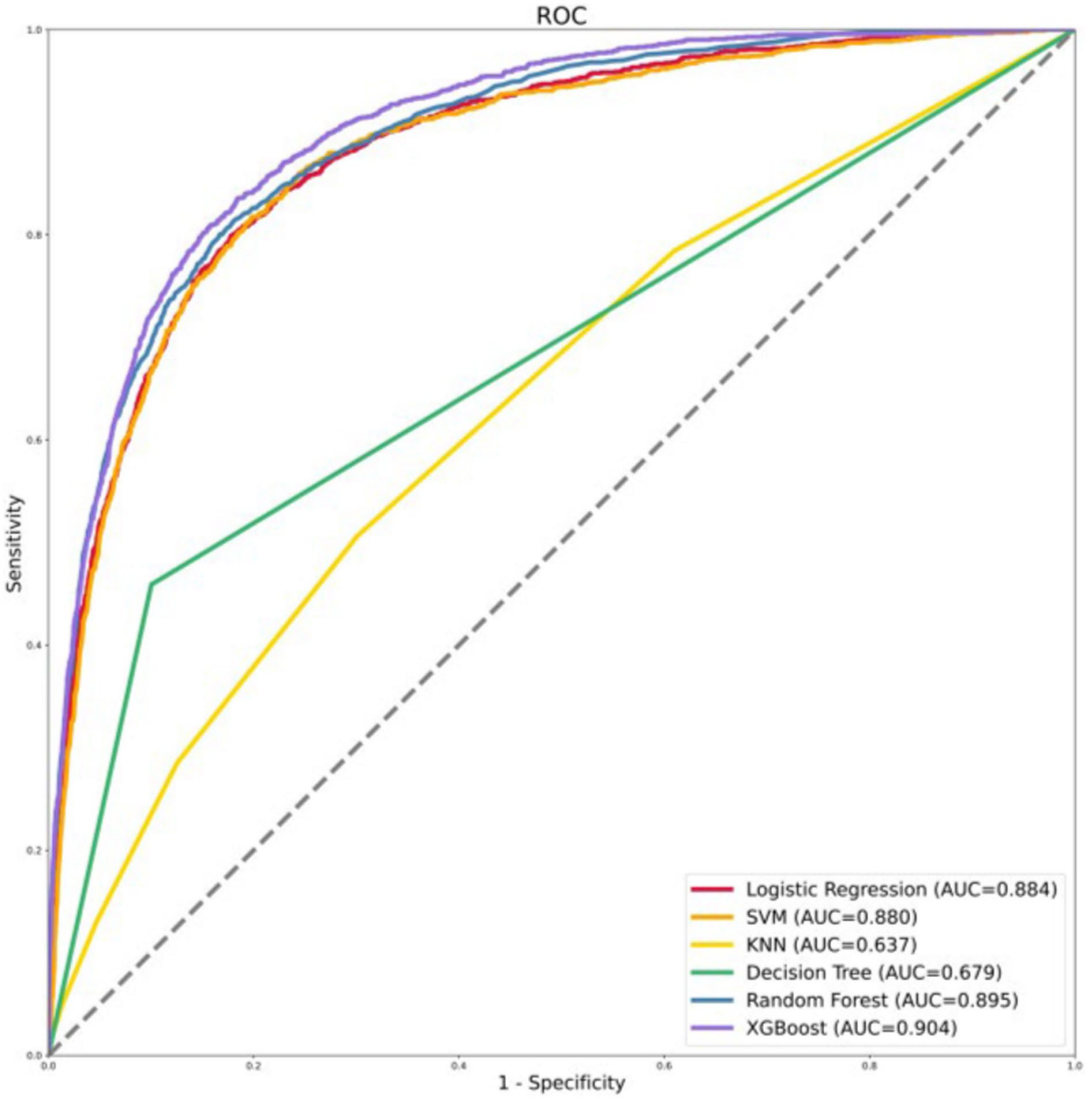
The ROC curves of six models.

In total, 53,150 patients were randomly divided into the training set (42,520, 80%) and validation set (10,630, 20%). We constructed six ML models, including SVM, KNN, XGBoost, RF, LG, and DT, to predict the onset of EN. The calculated sensitivity, specificity, accuracy, and AUC are presented in Table 2. In the validation, the AUC of the XGBoost model was 0.895, higher than other models (LR: 0.874; SVM: 0.868; KNN: 0.646; DT: 0.671; RF: 0.888, respectively) (Figure 2). The calibration curve and DCA for each model are depicted in Supplementary Figures S1, S2. The AUC of the XGBoost model outperformed the RF model without statistically significant (*p* = 0.373) (Supplementary Table S4). Nevertheless, the XGBoost model was superior to RF in consistency and clinical utility by observing the calibration curve and DCA. To sum up, the XGBoost model presented more accurate in predicting performance among the 6 models we developed.

**Table 2:** The evaluation of the six ML models predictive performance.

Models	AUC (95% CI)	Accuracy	Sensitivity	Specificity
κ-Nearest neighbor	0.637 (0.620–0.653)	0.867	0.506	0.699
Decision tree	0.679 (0.663–0.696)	0.841	0.459	0.900
Support vector machine	0.880 (0.868–0.892)	0.867	0.818	0.802
Logistic regression	0.884 (0.872–0.895)	0.895	0.799	0.822
Random forest	0.895 (0.883–0.906)	0.896	0.801	0.833
XGBoost	0.904 (0.893–0.915)	0.901	0.809	0.842

AUC, area under curve; CI, confidence interval; XGBoost, Extreme Gradient Boosting.

### 3.3. Model explainability

**Figure 3:**
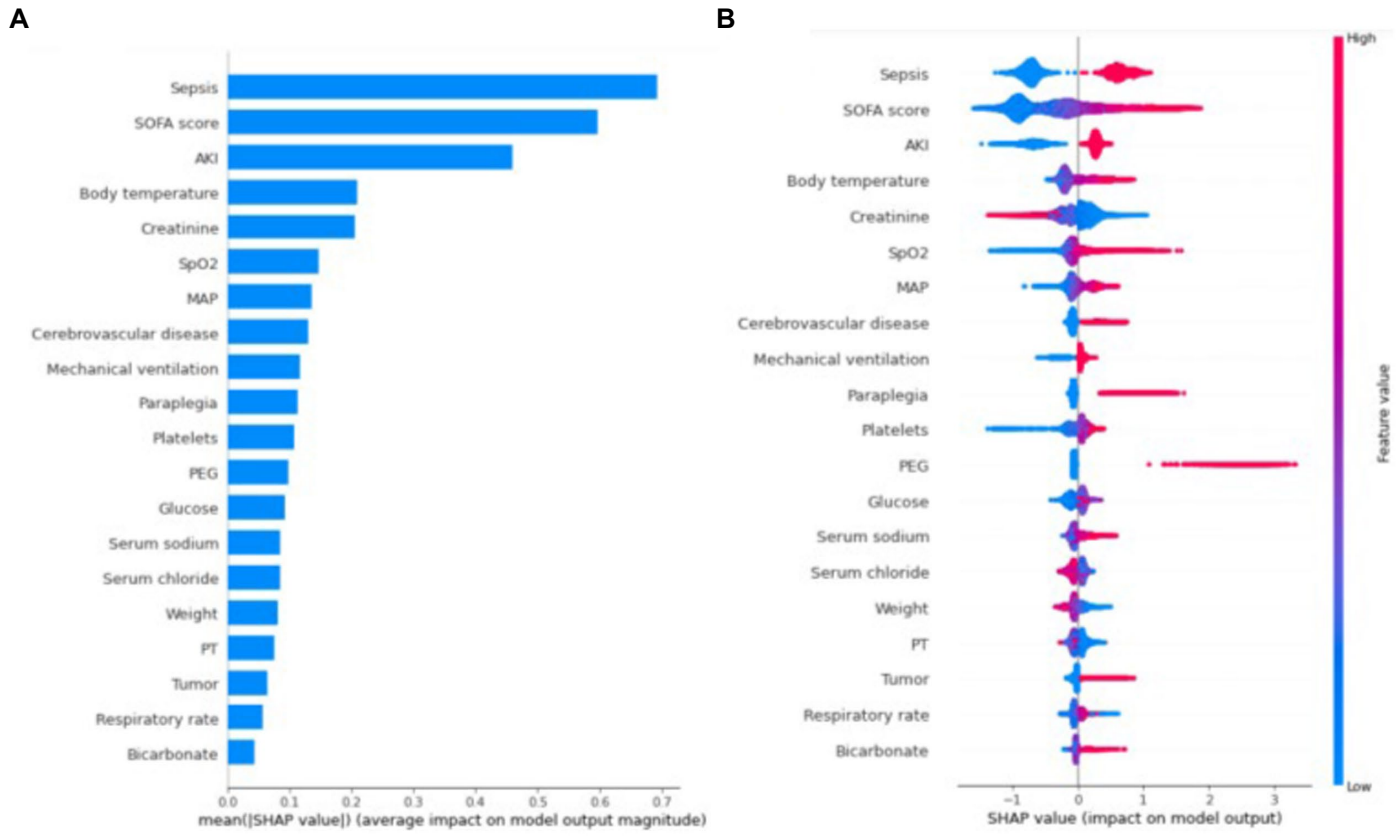
SHAP summary plot for the first 20 clinical features contributing to the XGBoost model. **(A)** The ranking of importance features in the XGBoost model. The matrix of SHAP summary plot describes the importance of each features in the development of the XGBoost model. **(B)** The attribute of each feature in the final model. The horizontal coordinate represents the SHA*p* value. Each line indicates a feature, and the high and low feature values are shown in red and blue, respectively. SOFA, sequential organ failure assessment; AKI, acute kidney injury; SpO2, oxygen saturation; MAP, mean arterial pressure; PEG, percutaneous endoscopic gastrostomy; PT, prothrombin time; SHAP, SHapley Additive explanation.

**Figure 4:**
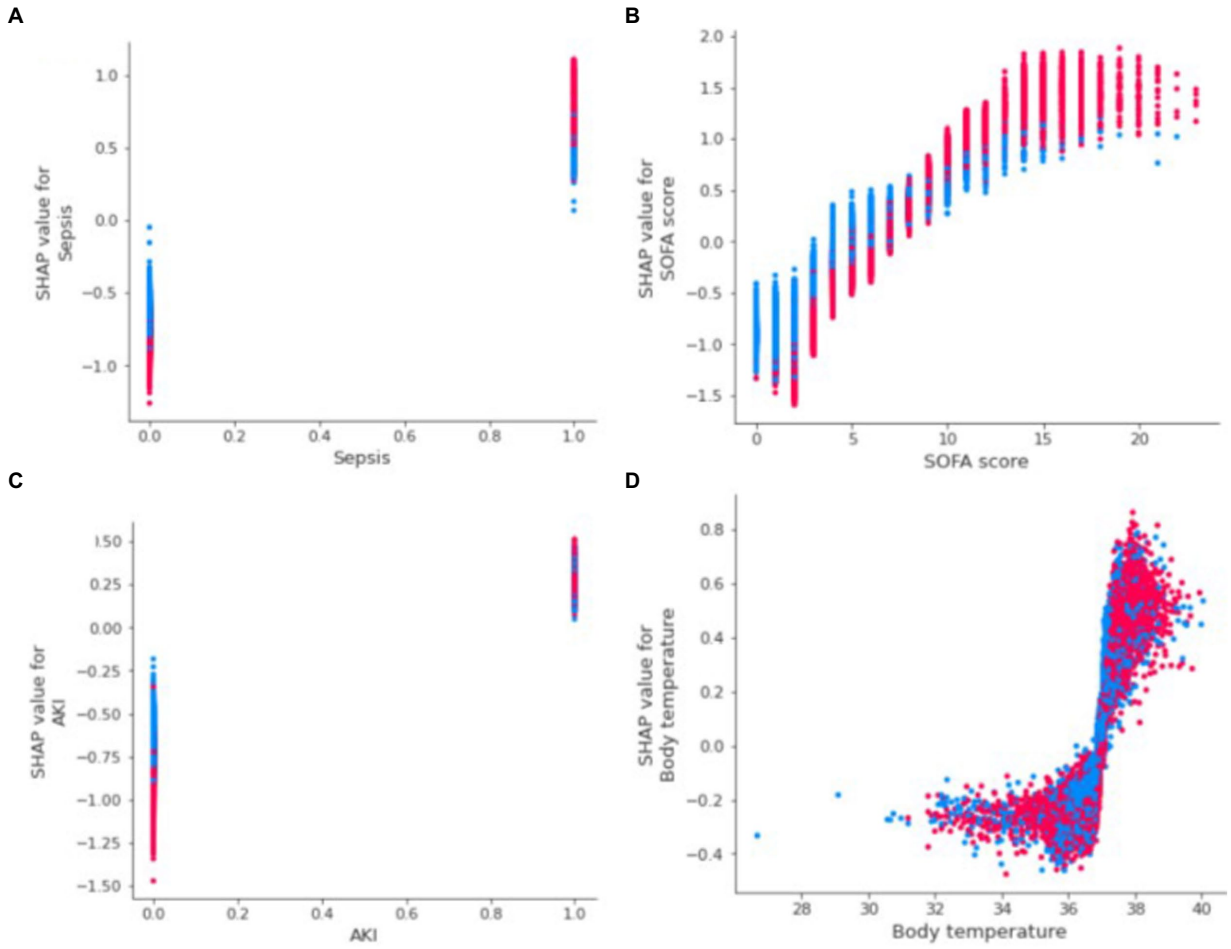
SHAP dependence plot of the first 4 variables influencing the XGBoost model. **(A)** Sepsis; **(B)** SOFA score; **(C)** AKI; **(D)** Body temperature. The probabilities of EN initiation increase when SHAP values of features exceed zero. AKI: acute kidney injury, SHAP: SHapley Additive explanation, SOFA: sequential organ failure assessment, XGBoost: eXtreme Gradient Boosting, EN: enteral nutrition.

**Figure 5:**
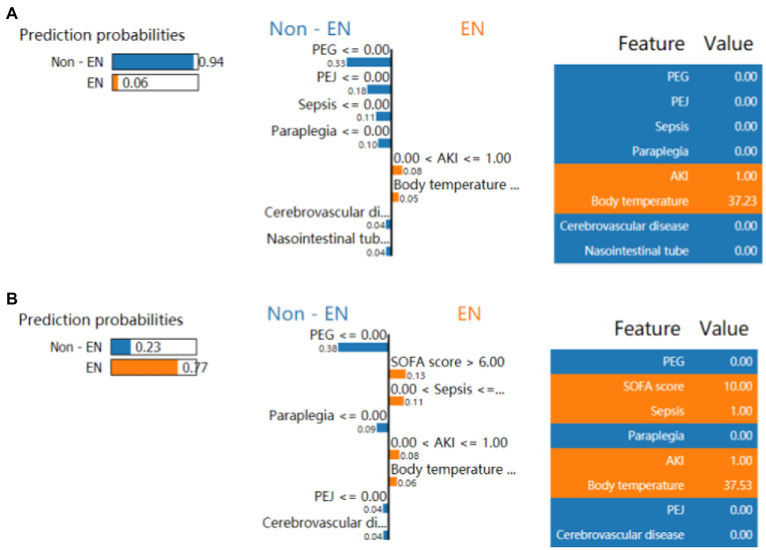
LIME algorithm for explaining individualized prediction. **(A, B)** Showed the risk prediction charts for the use of EN in critically ill patients or not. **(A)** Presented a Non-EN case with the LIME algorithm. **(B)** Presented a EN case with the LIME algorithm. The prediction probabilities by LIME are depicted in the left of the two screenshots. The first 8 features that significantly affected on using EN or not are presented in the middle sections from top to bottom. The weight (importance) of each feature in risk prediction is indicated per the bar’s length. The longer bar represents the more contribution to EN initiation or not. The critical values of these 8 features are shown on the right parts when they had the maximize effect on using EN or not. LIME, local interpretable model-agnostic explanations; EN, enteral nutrition; AKI, acute kidney injury; PN, parental nutrition; PEG, percutaneous endoscopic gastrostomy. PEJ, Percutaneous Endoscopic Jejunostomy; SOFA, sequential organ failure assessment.

Figure 3A shows the ranking of the importance of the features of the XGBoost model. The top three factors contributing to the output of the XGBoost model were sepsis, SOFA score, and AKI. In addition, it is depicted in Figure 3B how a single feature affected the predicted effect of the dependent variable in the model. Figure 4 reveals a detailed change trend of the top four variables that affect the model output. In order to understand how the model achieves individualized prediction, we extracted a sample from the validation set for interpretation (Figure 5). The patient was 43 years old and had chronic pulmonary disease, SOFA score of 3, and did not use EN. Likely, the predicted outcome of the XGBoost model was that the risk of not using EN was 94% (Figure 5A). The AKI and body temperature of 37.23°C contributed to the increased EN initiation rate. Factors that did not recommend EN were no PEG, no PEJ, no sepsis, no paraplegia, no cerebrovascular disease, or no nasointestinal tube. The other patient was a 34-year-old man and used EN. Likely, the predicted outcome of the XGBoost model was more inclined to implement EN. Factors that recommended EN were SOFA score of 10, sepsis, AKI, and body temperature of 37.53°C. No PEG, no paraplegia, no PEJ, or no cerebrovascular disease decreased EN initiation rate.

## 4. Discussion

In this study, we developed and validated the application of six machine learning methods for early evaluation of EN in ICU patients, and the XGBoost model showed the best accuracy. The ranking of feature importance and the influence of variables on XGBoost prediction were described per SHAP values. The top 4 significant factors were sepsis, SOFA score, AKI, and body temperature. We also used the LIME algorithm to perform the individualized predictions of XGBoost. Only 13.57% (7,210/53150) of patients used EN in this study. Early prediction of EN initiation may be helpful to improve EN implementation in ICU patients.

Based on the MIMIC-IV database, we developed and validated an effective tool for predicting EN initiation. XGBoost combines multiple weak classification tree models to establish an accurate prediction model. It offers the benefits of excellent generalization ability, automatic processing of missing values, flexibility and robustness, and fast operation speed. XGBoost, on the other hand, can solve this problem of overfitting or underfitting in ML models, which results from the low occurrence rate of the predicted events (the ratio of the number of events to the total number of samples is less than 1:10) (19). Choi et al. predicted refeeding hypophosphatemia, XGBoost showed higher accuracy than Logistic, Lasso, and Ridge pressures (20).

Additionally, explainable AI (XAI) was applied to facilitate users’ comprehension of the decision-making process of the models (21). In the present study, SHAP values and the LIME method were incorporated into the XGBoost model to attain the highest predicted accuracy and interpretability. Individual explanations by the LIME algorithm can provide evidence for the model’s prediction results and assist physicians in better use models when making decisions.

Our study found that sepsis was the strongest predictor of EN initiation. In sepsis, inflammatory mediators induce the catabolism of protein, fat, and carbohydrate stored in the body, which leads to severe energy shortage in patients. There are studies showing that early moderate EN decreases mortality and incidence of secondary inflammation in sepsis patients (22, 23). Several guidelines recommend that EN be initiated early and gradually after hemodynamic stability (3, 17). The SOFA score is used to determine the extent of major organ failure in critically ill patients, which is strongly associated with prognosis. In ranking the XGBoost model’s feature importance, the SOFA score was found to be the second key factor, consistent with previous findings (24). Increased nutritional intake can improve outcomes in patients with higher SOFA scores (24). In addition, AKI and body temperature were also important factors affecting EN initiation. The patients with AKI have not only abnormal metabolism of water and electrolyte but also carbohydrates, protein, and lipid. They are at high risk for malnutrition and muscle wastage (25). This study considered that the possibility of starting EN was greater, as the body temperature increase led to more body consumption. Unfortunately, a study showed that elevated body temperature was related to the failure of EN initiation in patients with severe acute pancreatitis (26). To investigate the relationship between body temperature and EN initiation, more studies should be performed. Interestingly, this study found that the placement of PEG is another predictor of EN initiation. ESPEN recommends that PEG is preferred for patients with prolonged EN (27).

This was, as far as we are aware, the first study to use the XGBoost model as a predictive tool in EN initiation. We are committed to opening the black box of ML models and using interpretable ML approaches (SHAP value and LIME algorithm) to better explain how predictors contribute to the model and how the model makes judgments. It is important to note that the study has limitations. We neglected to include the acute gastrointestinal injury grade, a crucial indicator of understanding gastrointestinal function, in the models’ development, because it was not reported in the MIMIC-IV database. These may have led to selected bias. This is a retrospective study. The patients had already been discharged from the hospital at the time of data extraction, and the use of EN had already been determined. Thus, we have no way of knowing if the EN was started according to the gold standard. Thirdly, XGBoost can explore the interaction between features, but not all interactions can be displayed in LIME. Finally, this study developed a model based on single-center data and only conducted internal validation. These findings may not generalize to other populations. More factors need to be included for further model optimization, and prospective multicenter studies should be performed to verify the external applicability of this model.

## 5. Conclusion

We developed and validated the application of six machine learning methods for early evaluation of EN in ICU patients, and the XGBoost model showed the best predictive performance. Sepsis, SOFA score, AKI, and body temperature were used as the four most important predictors of EN initiation.

## Data availability statement

Publicly available datasets were analyzed in this study. This data can be found here: https://physionet.org/content/mimiciv/1.0/.

## Ethics statement

Consent had access to MIMIC databases. MIMIC IV was set up with the approval of the Institutional Review Board at the Massachusetts Institute of Technology. All participant data were anonymized to safeguard their privacy. Due to the use of anonymized health records, ethical approval and informed consent were not required.

## Author contributions

Y-XW, X-LL, and X-FP contributed to the design and conceptualization of the study. X-LL and Y-XW extracted the data and analyzed the results. Y-XW and L-HZ drafted the manuscript. H-NL, X-ML, and WS contributed to the interpretation of the data. X-FP revised the manuscript. All authors read, commented, and approved the final manuscript.

## Conflict of interest

The authors declare that the research was conducted in the absence of any commercial or financial relationships that could be construed as a potential conflict of interest.

## Publisher’s note

All claims expressed in this article are solely those of the authors and do not necessarily represent those of their affiliated organizations, or those of the publisher, the editors and the reviewers. Any product that may be evaluated in this article, or claim that may be made by its manufacturer, is not guaranteed or endorsed by the publisher.

## Abbreviations

AKI: Acute kidney injury
AI: artificial intelligence
AUC: Area under curve
DCA: decision curve analysis
DT: Decision tree
EN: Enteral nutrition
ICU: Intensive care unit
KNN: κ-nearest neighbor
LIME: Local Interpretable Model-Agnostic Explanations
LR: Logistic regression
MAP: Mean arterial pressure
MI: multiple imputation
MIMIC-IV: Medical Information Mart for Intensive Care IV
PEG: percutaneous endoscopic gastrostomy
PEJ: percutaneous endoscopic jejunostomy
RF: Random Forest
SHAP: SHapley Additive exPlanation
SOFA: Sequential Organ Failure Assessment
SpO_2_: Oxygen saturation
SVM: Support vector machine
TRIPOD: Transparent Reporting of a multivariable prediction model for Individual Prognosis or Diagnosis Statement
XAI: artificial intelligence
XGBoost: Extreme Gradient Boost
