# Supplementary material for: Machine learning algorithms assist early evaluation of enteral nutrition in ICU patients
Source: Front Nutr. 2023 Apr 14;10:1060398. doi: 10.3389/fnut.2023.1060398 (PMC10140307; doi:10.3389/fnut.2023.1060398)
Supplement: Supplementary file 1 [file Data_Sheet_1.docx]

**Table S1 The variables of laboratory findings**

| **Item** | **Variables** |
| --- | --- |
| **Laboratory findings** | hematocrit, hemoglobin, white blood cell, bicarbonate, serum chloride, serum sodium, serum potassium, serum calcium, anion gap, glucose, serum creatinine, platelets, prothrombin time, international normalized ratio, partial thromboplastin time, blood urea nitrogen and the estimated glomerular filtration rate |

**Table S2. Missing number (%) for variables**

| **Variables** | **Missing number (%)** |
| --- | --- |
| **Age** | 0(0) |
| **Sex, male** | 0(0) |
| **Weight** | 1082(2.0%) |
| **Ethnicity** | 0(0) |
| **Congestive heart failure** | 0(0) |
| **Peptic ulcer disease** | 0(0) |
| **Myocardial infarction** | 0(0) |
| **Peripheral vascular disease** | 0(0) |
| **Diabetes** | 0(0) |
| **Dementia** | 0(0) |
| **Chronic pulmonary disease** | 0(0) |
| **Rheumatic disease** | 0(0) |
| **Cerebrovascular disease** | 0(0) |
| **Cancer** | 0(0) |
| **Paraplegia** | 0(0) |
| **Liver disease** | 0(0) |
| **Intestinal fistula** | 0(0) |
| **Short bowel syndrome** | 0(0) |
| **Acute pancreatitis** | 0(0) |
| **Abdominal Hypertension** | 0(0) |
| **AKI** | 0(0) |
| **Renal disease** | 0(0) |
| **Aids** | 0(0) |
| **Sepsis** | 0(0) |
| **Heart rate** | 84(0.1%) |
| **MAP** | 119(0.2%) |
| **Respiratory rate** | 138(0.2%) |
| **Body temperature** | 1733(3.2%) |
| **SpO_2_** | 130(0.2%) |
| **Hematocrit** | 442(0.8%) |
| **Hemoglobin** | 486(0.9%) |
| **Platelets** | 479(0.9%) |
| **WBC** | 478(0.9%) |
| **BUN** | 409(0.7%) |
| **Anion gap** | 673(1.3%) |
| **INR** | 5281(9.9%) |
| **Serum Creatinine** | 406(0.7%) |
| **Serum glucose** | 803(1.5%) |
| **Serum calcium** | 5102(9.6%) |
| **Serum chloride** | 607(1.1%) |
| **Bicarbonate** | 619(1.2%) |
| **Serum potassium** | 640(1.2%) |
| **Serum sodium** | 612(1.2%) |
| **PT** | 5280(9.9%) |
| **PTT** | 5572(10.5%) |
| **eGFR** | 0(0) |
| **Dialysis** | 0(0) |
| **Vasopressors use** | 0(0) |
| **Mechanical ventilation** | 0(0) |
| **PEG** | 0(0) |
| **PEJ** | 0(0) |
| **Nasogastric tube** | 0(0) |
| **Nasointestinal tube** | 0(0) |
| **SOFA score** | 0(0) |
| **SAPS II score** | 0(0) |

Abbreviations: Aids: acquired immune deficiency syndrome, AKI: acute kidney injury, MAP: mean arterial pressure, SpO_2_: oxygen saturation, WBC: white blood cell, BUN: blood urea nitrogen, INR: international normalized ratio, PT: prothrombin time, PTT: partial thromboplastin time, eGFR: estimated glomerular filtration rate, PEG: percutaneous endoscopic gastrostomy, PEJ: Percutaneous Endoscopic Jejunostomy, SOFA: sequential organ failure assessment, SAPS II: Simplified Acute Physiology Score II.

**Table S3. Comparison of baseline characteristics between the training and test sets**

| **Variables** | **Total**  **(n =53150)** | **Training set**  **(n =42520)** | **Test set**  **(n =10630)** | **P value** |
| --- | --- | --- | --- | --- |
| **Age (years)** | 66.76 [54.49, 78.24] | 66.74 [54.42, 78.17] | 66.82 [54.75, 78.48] | 0.215 |
| **Sex, male, n (%)** | 29797 (56.1) | 23830 (56.0) | 5967 (56.1) | 0.877 |
| **Weight (kg)** | 78.45 [65.90, 93.10] | 78.40 [65.90, 93.00] | 78.50 [65.80, 93.50] | 0.898 |
| **Ethnicity, n (%)** |  |  |  | 0.915 |
| White | 35668 (67.1) | 28524 (67.1) | 7144 (67.2) |  |
| Black | 4874 (9.2) | 3907 (9.2) | 967 (9.1) |  |
| Other | 12608 (23.7) | 10089 (23.7) | 2519 (23.7) |  |
| **Myocardial infarction, n (%)** | 8531 (16.1) | 6839 (16.1) | 1692 (15.9) | 0.686 |
| **Congestive heart failure, n (%)** | 12622 (23.7) | 10144 (23.9) | 2478 (23.3) | 0.242 |
| **Peripheral vascular disease, n (%)** | 5820 (11.0) | 4645 (10.9) | 1175 (11.1) | 0.715 |
| **Cerebrovascular disease, n (%)** | 8539 (16.1) | 6821 (16.0) | 1718 (16.2) | 0.775 |
| **Dementia, n (%)** | 1930 (3.6) | 1531 (3.6) | 399 (3.8) | 0.469 |
| **Chronic pulmonary disease, n (%)** | 12398 (23.3) | 9864 (23.2) | 2534 (23.8) | 0.167 |
| **Rheumatic disease, n (%)** | 1717 (3.2) | 1361 (3.2) | 356 (3.3) | 0.458 |
| **Liver disease, n (%)** | 5766 (10.8) | 4666 (11.0) | 1100 (10.3) | 0.066 |
| **Intestinal fistula, n (%)** | 170 (0.3) | 140 (0.3) | 30 (0.3) | 0.501 |
| **Short bowel syndrome, n(%)** | 40 (0.1) | 35 (0.1) | 5 (0.0) | 0.323 |
| **Acute pancreatitis, n (%)** | 907 (1.7) | 726 (1.7) | 181 (1.7) | 1.000 |
| **Abdominal Hypertension, n (%)** | 1144 (2.2) | 955 (2.2) | 189 (1.8) | 0.003 |
| **Peptic ulcer disease, n (%)** | 1457 (2.7) | 1173 (2.8) | 284 (2.7) | 0.647 |
| **Diabetes, n (%)** | 14613 (27.5) | 11646 (27.4) | 2967 (27.9) | 0.286 |
| **Paraplegia, n (%)** | 2748 (5.2) | 2201 (5.2) | 547 (5.1) | 0.918 |
| **Renal disease, n (%)** | 9386 (17.7) | 7561 (17.8) | 1825 (17.2) | 0.142 |
| **Tumor, n (%)** | 7723 (14.5) | 6175 (14.5) | 1548 (14.6) | 0.929 |
| **Aids, n (%)** | 284 (0.5) | 225 (0.5) | 59 (0.6) | 0.800 |
| **AKI,n(%)** | 29551 (55.6) | 23689 (55.7) | 5862 (55.1) | 0.298 |
| **Sepsis, n (%)** | 23901 (45.0) | 19128 (45.0) | 4773 (44.9) | 0.884 |
| **Heart rate (beats/minute)** | 82.83 [73.25, 94.16] | 82.86 [73.24, 94.17] | 82.69 [73.40, 94.11] | 0.817 |
| **MAP (mmHg)** | 77.52 [71.27, 85.61] | 77.52 [71.27, 85.61] | 77.56 [71.26, 85.62] | 0.77 |
| **Respiratory rate (beats/minute)** | 18.38 [16.39, 20.96] | 18.39 [16.40, 20.97] | 18.36 [16.35, 20.91] | 0.258 |
| **Body temperature (°C)** | 36.81 [36.59, 37.07] | 36.81 [36.58, 37.07] | 36.81 [36.59, 37.07] | 0.231 |
| **SpO_2_ (%)** | 97.07 [95.69, 98.36] | 97.07 [95.69, 98.36] | 97.08 [95.71, 98.36] | 0.565 |
| **Hematocrit (%)** | 35.20 [31.00, 39.60] | 35.20 [31.00, 39.60] | 35.20 [31.00, 39.60] | 0.911 |
| **Hemoglobin (g/dL)** | 11.70 [10.20, 13.20] | 11.70 [10.20, 13.20] | 11.60 [10.20, 13.20] | 0.899 |
| **Platelets (K/uL)** | 210.00 [158.00, 275.00] | 211.00 [158.00, 275.00] | 210.00 [158.00, 274.00] | 0.693 |
| **WBC (K/uL)** | 12.30 [8.80, 16.70] | 12.20 [8.80, 16.70] | 12.30 [8.90, 16.80] | 0.139 |
| **Bicarbonate (mmol/L)** | 24.00 [22.00, 27.00] | 24.00 [22.00, 27.00] | 24.00 [22.00, 27.00] | 0.243 |
| **Anion gap (mEq/L)** | 15.00 [13.00, 18.00] | 15.00 [13.00, 18.00] | 15.00 [13.00, 18.00] | 0.493 |
| **BUN (mg/dL)** | 19.00 [14.00, 30.00] | 19.00 [14.00, 30.00] | 19.00 [14.00, 30.00] | 0.609 |
| **Serum calcium (mg/dL)** | 8.60 [8.10, 9.00] | 8.60 [8.10, 9.00] | 8.60 [8.10, 9.00] | 0.663 |
| **Serum sodium (mEq/L)** | 140.00 [137.00, 142.00] | 140.00 [137.00, 142.00] | 140.00 [137.00, 142.00] | 0.157 |
| **Serum chloride (mEq/l)** | 106.00 [102.00, 109.00] | 106.00 [102.00, 109.00] | 106.00 [102.00, 109.00] | 0.145 |
| **Serum potassium (mEq/L)** | 4.40 [4.00, 4.80] | 4.40 [4.00, 4.80] | 4.40 [4.00, 4.80] | 0.763 |
| **Creatinine (mg/dL)** | 1.00 [0.80, 1.40] | 1.00 [0.80, 1.40] | 1.00 [0.80, 1.40] | 0.330 |
| **Glucose (mg/dL)** | 137.00 [113.00, 178.00] | 137.00 [113.00, 178.00] | 137.00 [113.00, 178.00] | 0.667 |
| **INR** | 1.30 [1.10, 1.50] | 1.30 [1.10, 1.50] | 1.30 [1.10, 1.50] | 0.655 |
| **PT (s)** | 14.00 [12.40, 16.60] | 14.00 [12.40, 16.60] | 14.00 [12.40, 16.60] | 0.522 |
| **PTT (s)** | 31.30 [27.50, 40.00] | 31.30 [27.50, 40.00] | 31.30 [27.50, 39.70] | 0.652 |
| **eGFR, ml/min/1.73 m^2^** | 1.01 [0.81, 1.06] | 1.01 [0.81, 1.06] | 1.01 [0.81, 1.06] | 0.664 |
| **Dialysis, n (%)** | 1668 (3.1) | 1337 (3.1) | 331 (3.1) | 0.896 |
| **Vasopressors use, n (%)** | 2004 (3.8) | 1598 (3.8) | 406 (3.8) | 0.789 |
| **Parental Nutrition, n(%)** | 885 (1.7) | 711 (1.7) | 174 (1.6) | 0.832 |
| **Mechanical ventilation, n (%)** | 38366 (72.2) | 30717 (72.2) | 7649 (72.0) | 0.566 |
| **PEG, n (%)** | 934 (1.8) | 762 (1.8) | 172 (1.6) | 0.238 |
| **PEJ, n (%)** | 66 (0.1) | 52 (0.1) | 14 (0.1) | 0.926 |
| **Nasogastric tube, n (%)** | 164 (0.3) | 139 (0.3) | 25 (0.2) | 0.153 |
| **Nasointestinal tube, n (%)** | 47 (0.1) | 39 (0.1) | 8 (0.1) | 0.743 |
| **SOFA** **score** | 4.00 [2.00, 6.00] | 4.00 [2.00, 6.00] | 4.00 [2.00, 6.00] | 0.496 |
| **SAPS II** **score** | 33.00 [25.00, 42.00] | 33.00 [25.00, 42.00] | 33.00 [25.00, 42.00] | 0.978 |

Abbreviations: Aids: acquired immune deficiency syndrome, AKI: acute kidney injury, MAP: mean arterial pressure, SpO_2_: oxygen saturation, WBC: white blood cell, BUN: blood urea nitrogen, INR: international normalized ratio, PT: prothrombin time, PTT: partial thromboplastin time, eGFR: estimated glomerular filtration rate, PEG: percutaneous endoscopic gastrostomy, PEJ: Percutaneous Endoscopic Jejunostomy, SOFA: sequential organ failure assessment, SAPS II: Simplified Acute Physiology Score II.

**Table S4. The difference of AUCs between the six models using the DeLong test**

|  | **XGBoost** | **RF** | **LR** | **SVM** | **KNN** | **DT** |
| --- | --- | --- | --- | --- | --- | --- |
| **XGBoost** |  |  |  |  |  |  |
| **RF** | 0.373 |  |  |  |  |  |
| **LR** | 0.039 | 0.322 |  |  |  |  |
| **SVM** | 0.025 | 0.100 | 0.573 |  |  |  |
| **KNN** | <0.001 | <0.001 | <0.001 | <0.001 |  |  |
| **DT** | <0.001 | <0.001 | <0.001 | <0.001 | <0.001 |  |

Abbreviations: AUC: area under curve; XGBoost: eXtreme Gradient Boosting; RF: Random Forest; SVM: Support Vector Machine; LR: logistic regression; KNN: k-Nearest Neighbors; DT: Decision Tree

**Figure titles and legends**

**Figure S1** The calibration curves for the six models

**Figure S2** The clinical impact curves for the six models


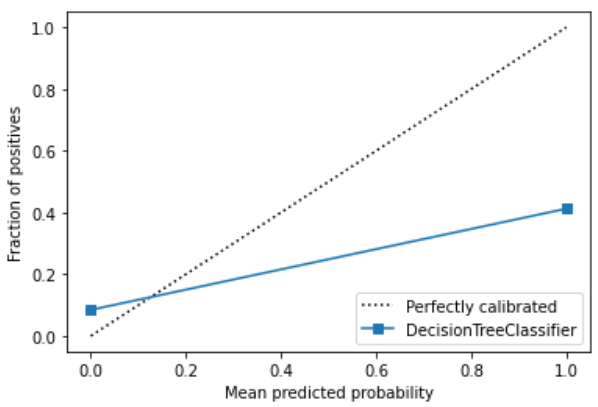

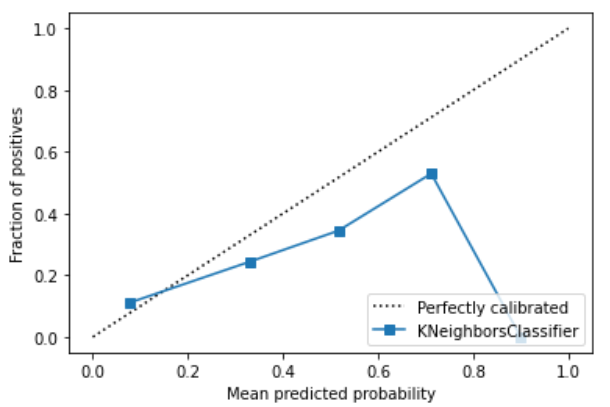


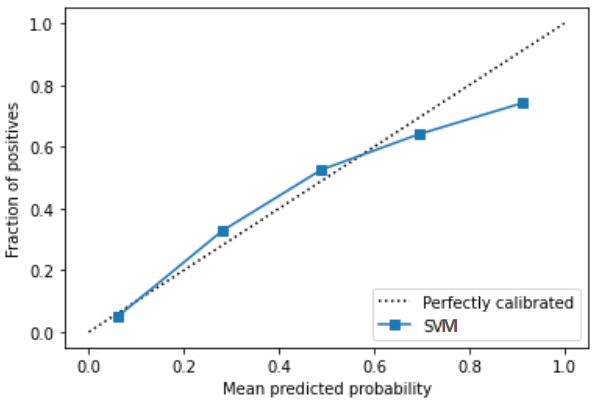

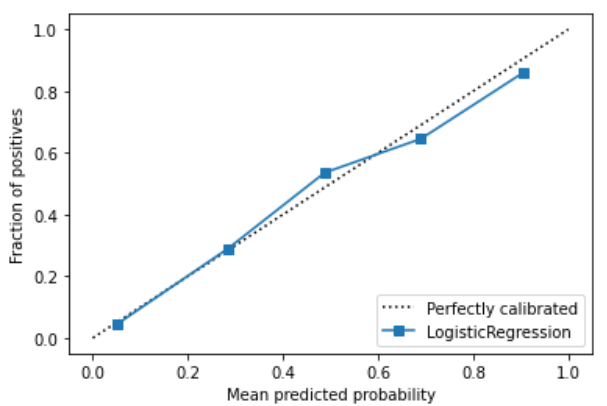

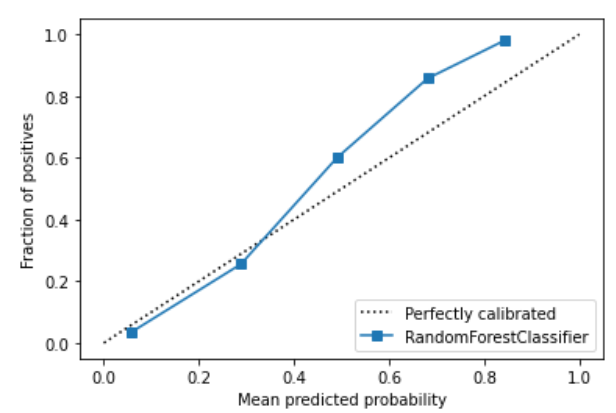

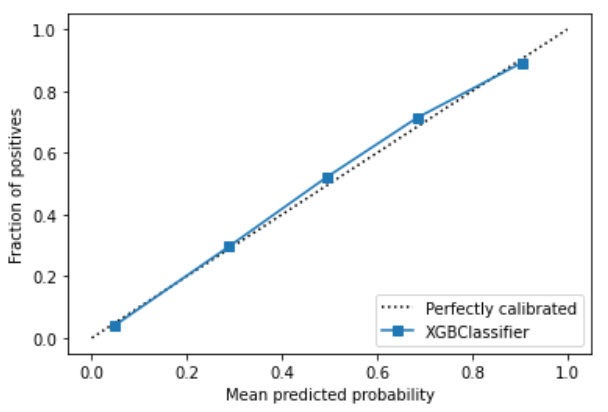


**Figure S1** The calibration curves for the six models

Abbreviations: SVM: support vector machine.

**
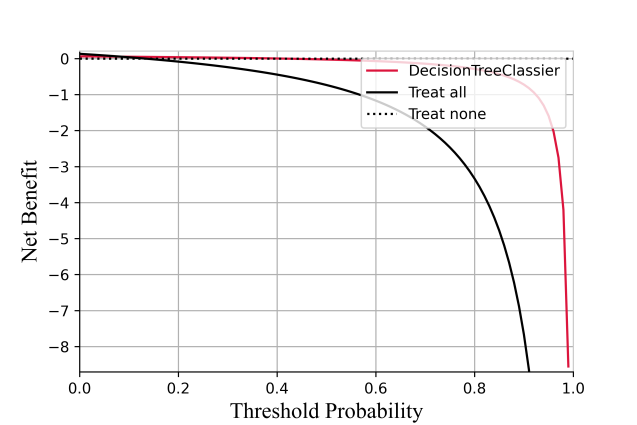

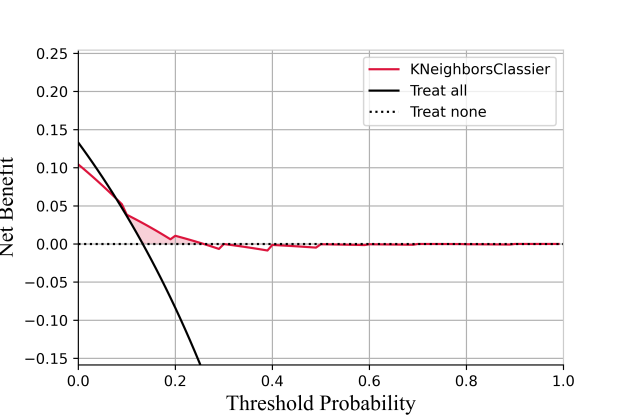
**

**
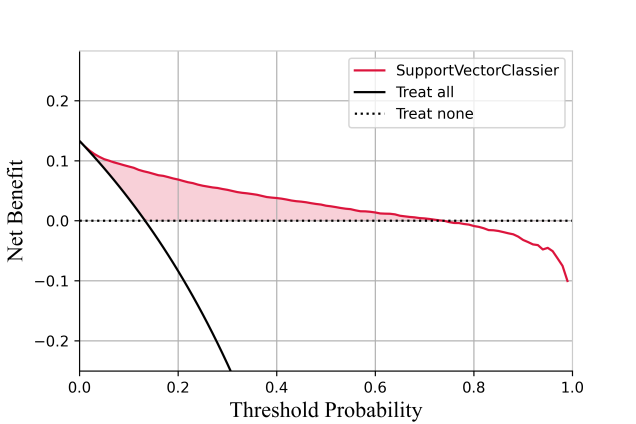

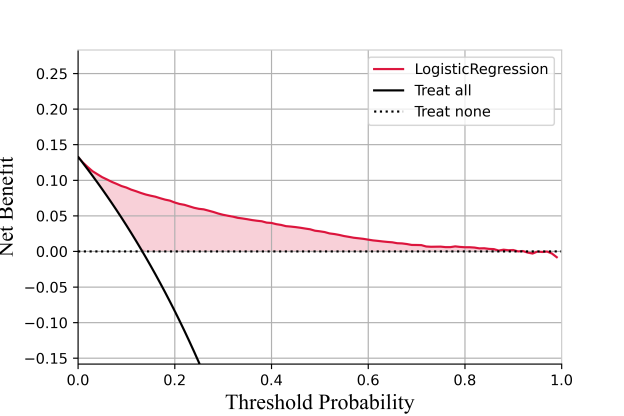

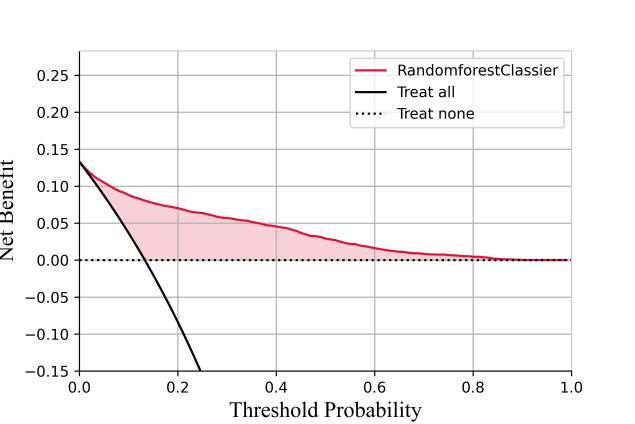

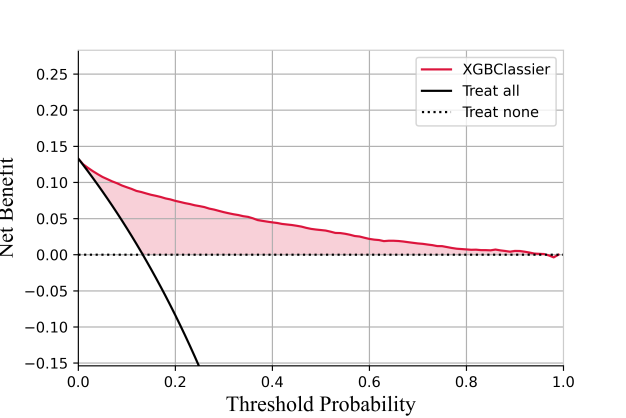
**

**Figure S2** The clinical impact curves for the six models
